# Neuromuscular activation pattern of lower extremity muscles during pedaling in cyclists with single amputation of leg and with two legs: a case study

**DOI:** 10.1186/s13104-020-05144-9

**Published:** 2020-06-22

**Authors:** Kohei Watanabe, Yuta Yamaguchi, Wataru Fukuda, Sho Nakazawa, Taishi Kenjo, Tetsunari Nishiyama

**Affiliations:** 1 Laboratory of Neuromuscular Biomechanics, School of International Liberal Studies, Chukyo University, Yagotohonmachi, Showa-ku, Nagoya, 466-8666 Japan; 2 Nippon Sport Science University, Tokyo, Japan; 3 Yokohama Sports Medical Center, Yokohama, Japan; 4 Japan Para-Cycling Federation, Tokyo, Japan

**Keywords:** Electromyography, Paralympic, Quadriceps muscle, Central nervous system, Athletes, Rectus femoris

## Abstract

**Objective:**

In Para-cycling competitions, cyclists with amputation of one-leg and no prosthesis, i.e., Division Cycle, Sport Class C2, perform pedaling movement on bicycle by unilateral leg. The purpose of this study was to describe neuromuscular activation of lower extremity muscles in two cyclists with single leg amputation and one cyclist with two legs during pedaling. We compared averaged rectified values (ARV) of surface electromyography for lower extremity muscles during crank cycle for two single leg cyclists with one cyclist with two legs at 65%, 80%, and 95% of VO_2_ max.

**Results:**

Characteristic features of cyclists with single amputation of leg were increases in ARV for proximal region of the rectus femoris muscle in first half of pulling phase, increases in ARV for the biceps femoris muscle in first half of pulling phase, and increases in ARV for the medial gastrocnemius muscle in pulling phase. These findings in this study suggest that cyclists with single amputation of leg use characteristic neuromuscular coordination in the muscles contributing to hip and knee flexion joint moments during pulling phase and this may be the strategy in cyclists with single amputation of leg to compensate lack of hip and/or knee extension torque from contralateral leg.

## Introduction

Pedaling movement on bicycle mainly consists of pushing and pulling actions by lower extremity joints and related muscles to rotate cranks via pedals. Since two cranks are linked on a bicycle, pushing and pulling actions by two legs would be well coordinated in cyclists [2, 10]. In Para-cycling competitions, cyclists with amputation of one-leg and no prosthesis, i.e., Division Cycle, Sport Class C2, perform pedaling movement on bicycle by unilateral leg. Their pedaling skill should not be comparable with those by two legs and different neuromuscular coordination strategy would be used in cyclists with single amputation of leg. In the case of one-leg pedaling movements, contribution of pulling action to rotation of cranks could be greater than two-leg cycling because pushing action produce the power to rotate crank during half of crank cycle [3]. Hasson et al. [5] reported that a single practice session of one-leg pedaling to direct cyclist’s applied pedal force perpendicular to the crank arm increased hip and knee flexion joint torques [5]. Therefore, it is reasonable to assume that cyclists with single amputation of leg emphasize to activate hip and/or knee flexor muscles during pulling phase. Understanding of the pedaling technique related with neuromuscular activation would provide helpful knowledges to improve pedaling efficiency and then performance in competitions [1].

The purpose of this study was to describe neuromuscular activation of lower extremity muscles in two cyclists with single leg amputation and one cyclist with two legs during pedaling. This is case study because of small number of samples.

## Main text

### Methods

#### Participants

Two cyclists with single amputation of leg (CS1, male; CS2, female) who participate Sport Class C2 in competitive Para-cycling and one cyclist with two legs (CT, male) were recruited in this study. These cyclists participate world championships of Para-cycling (Sport Class C2 for CS1 and CS2 and tandem pilot of visual impaired cyclist for CT) and 3, 5, and 12 (The tandem competition of para-cycling was 2 years) years of competitive cycling experiences for CS1, CS2, and CT. The main competition results from 2016 to 2018 in these participants were 3rd place in UCI Track cycling World Championships for CS1, 3rd place in Asian athletics championships for CS2, and first place in Asian athletics championships, and first place in Asian athletics championships (participate as a tandem pilot of visual impaired) for CT. They gave written informed consent for the study after receiving a detailed explanation of the purposes, potential benefits, and risks associated with participation. All procedures used in this study were in accordance with the Declaration of Helsinki and approved by the Ethics Committee at Nippon Sports Science University (No. 018-H069).

#### Experimental design

Participants performed maximum exercise tolerance test to estimate maximum oxygen consumption (VO_2_ max). In same day of the maximum exercise tolerance test, submaximal pedaling exercises were performed at various relative workloads to measure surface electromyography (sEMG) from lower extremity muscles on an electrically braked cycle ergometer with saddle and handle positions that subjects usually used. Submaximal pedaling exercises were conducted 3 h after the maximum exercise tolerance test.

From the results of the maximum exercise tolerance test, we calculated workloads at 65%, 80%, and 95% of VO_2_ max for each participant and used for submaximal pedaling test. Participants were instructed to maintain their optimal pedaling cadence for 2 min for 65% and 80% of VO_2_ max and 1–2 min for 95% of VO_2_ max. Same pedaling cadences were used among three workloads within each participant. Three workloads were applied in order of 65%, 80%, and 95% of VO_2_ max with at least of 5 min of rest among the exercises with different workloads.

#### Surface electromyography

During submaximal pedaling exercises, sEMG was recorded from RF, vastus lateralis (VL), biceps femoris (BF), medial gastrocnemius (MG), and tibialis anterior (TA) muscles using wireless sEMG device (Sessantaquattro, OT Bioelettronica, Torino, Italy) with 1000 Hz of sampling frequency and 256 of amplitude gain. These muscles have been often interested as the muscles which contributes to pedaling movements in the previous studies [7, 8]. We used bipolar electrodes with 15 mm of diameter of gel type detection area (CDE, OT Bioelettronica, Torino, Italy) and 20 mm of inter-electrode distance. Electrode locations for VL, BF, MG, and TA muscles were determined by the recommendations from SENIAM [6]. For RF muscle, we recorded proximal (RFp) and distal regions (RFd) of the muscle. Recently, we reported that proximal regions of the rectus femoris (RF) muscle preferentially contributes to hip flexion joint moment [12] and this region-specific functional role within this muscle had been confirmed during pedaling movement [11]. So, this study applied this separated recording of surface EMG from RF muscle to assess the neuromuscular activation of hip flexor muscle. Electrodes for proximal and distal regions of RF muscle were placed at one-sixth length of the distance between the anterior superior iliac spine and superior edge of patella from the anterior superior iliac spine and superior edge of patella, respectively.

#### Data analysis and statistics

Averaged rectified value (ARV) of sEMG amplitude was calculated every 10° of crank angle for 2 min for 65% and 80% of VO_2_ max and 1–2 min for 95% of VO_2_ max. In this study, top dead of center in crank cycle was defined as 0°. Crank angle was calculated from a motion capture system (Vicon, Vicon Motion System Ltd. Oxford, UK). Three dimensional coordinates of reflective markers on pedal center and crank center were captured by eight infrared cameras at 200 Hz of sampling rate. Coordinates of these markers on sagittal plane were filtered by Butterworth low-pass digital filter with 8 Hz. From the filtered data, angles relative to a perpendicular line and the line between pedal center and crank center were calculated as crank angle.

Relationship between crank angle and ARV [13] at various workload was represented for each muscle and each participant for the further analyses. For each participant, the effect of increase in workload on ARV were qualitatively assessed to estimate the role of each muscle during pedaling performance.

### Results

#### Rectus femoris proximal region (RFp)

**Fig. 1:**
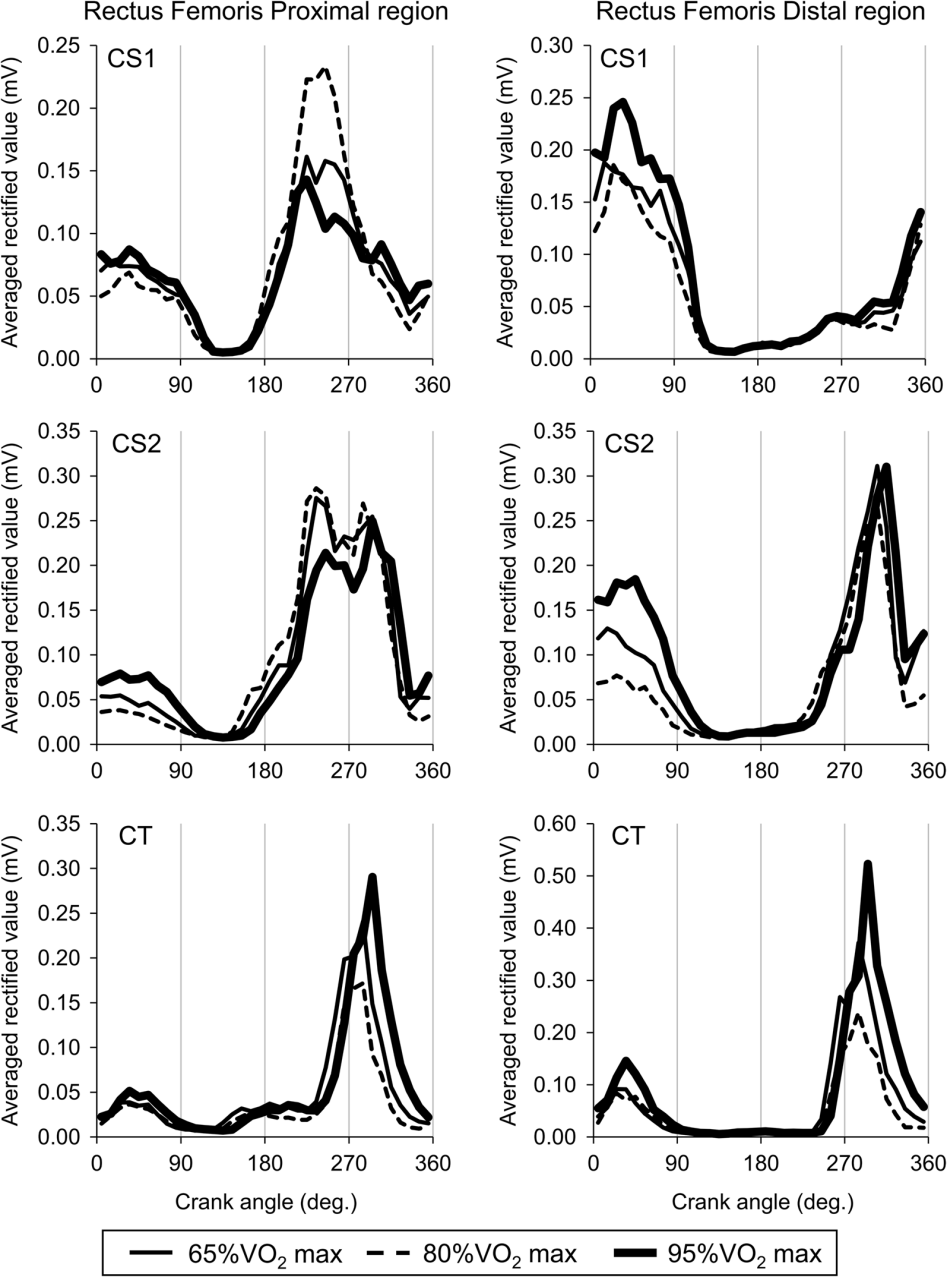
Averaged rectified values of surface electromyography during a crank cycle for proximal region of the rectus femoris muscle (Left panels) and distal region of the rectus femoris muscle (Right panels) at various workloads. CS1 and CS2, cyclists with single amputation of leg; CT, cyclist with two legs

CS1 and CS2 showed increases in ARV from 0° to 90° of crank angle and from 180° to 360° of crank angle and latter activations were greater than former activations (CS1 and CS2 in left panels of Fig. 1). In CT, an increase in ARV around 270° of crank angle with shorter duration and this is seemed to be increased with an increase in workload (CT in left panels of Fig. 1).

#### Rectus femoris distal region (RFd)

In CS1 and CS2, an increase in ARV was found from 270° to 90° of crank angle and this is increased with an increased in workload (CS1 in right panels of Fig. 1). CS2 and CT showed increases in ARV from 270° to 360° of crank angle were increased with an increase in workload (CT in right panels of Fig. 1).

#### Vastus lateralis (VL)

**Fig. 2:**
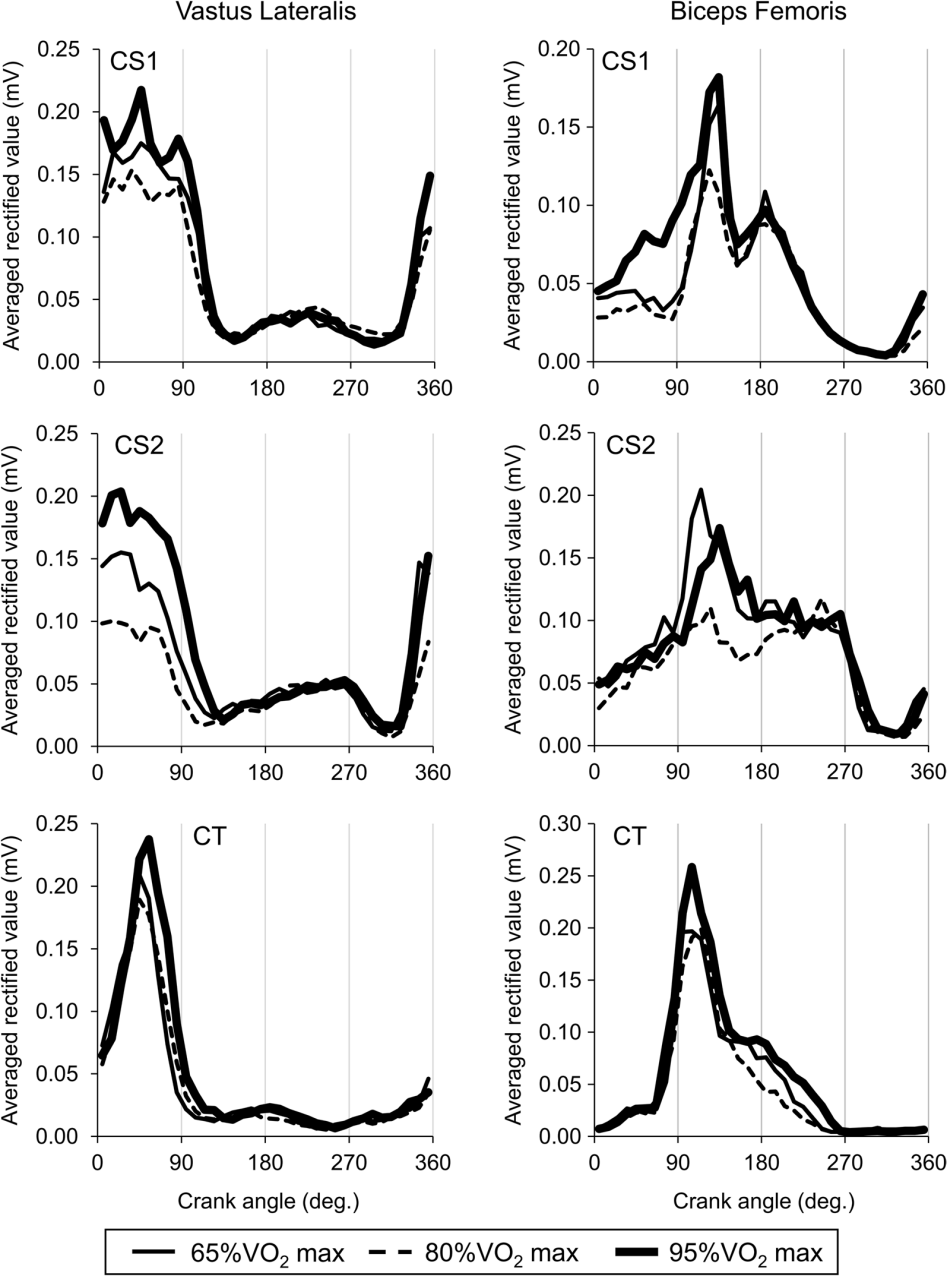
Averaged rectified values of surface electromyography during a crank cycle for the vastus lateralis muscle (Left panels) and the bicep femoris muscle (Right panels) at various workloads. CS1 and CS2, cyclists with single amputation of leg; CT, cyclist with two legs

For all participants, common patterns were represented, i.e., increases in ARV from before 0° to 90° of crank angle (Left panels of Fig. 2).

#### Biceps femoris (BF)

Increase in ARV were found from around 0° to 270° of crank angle for all participants (Right panels of Fig. 2). For CS1 and CS2, greater activations were shown from 180° to 270° of crank angle when compared with CT (Right panels of Fig. 2).

#### Medial gastrocnemius (MG)

**Fig. 3:**
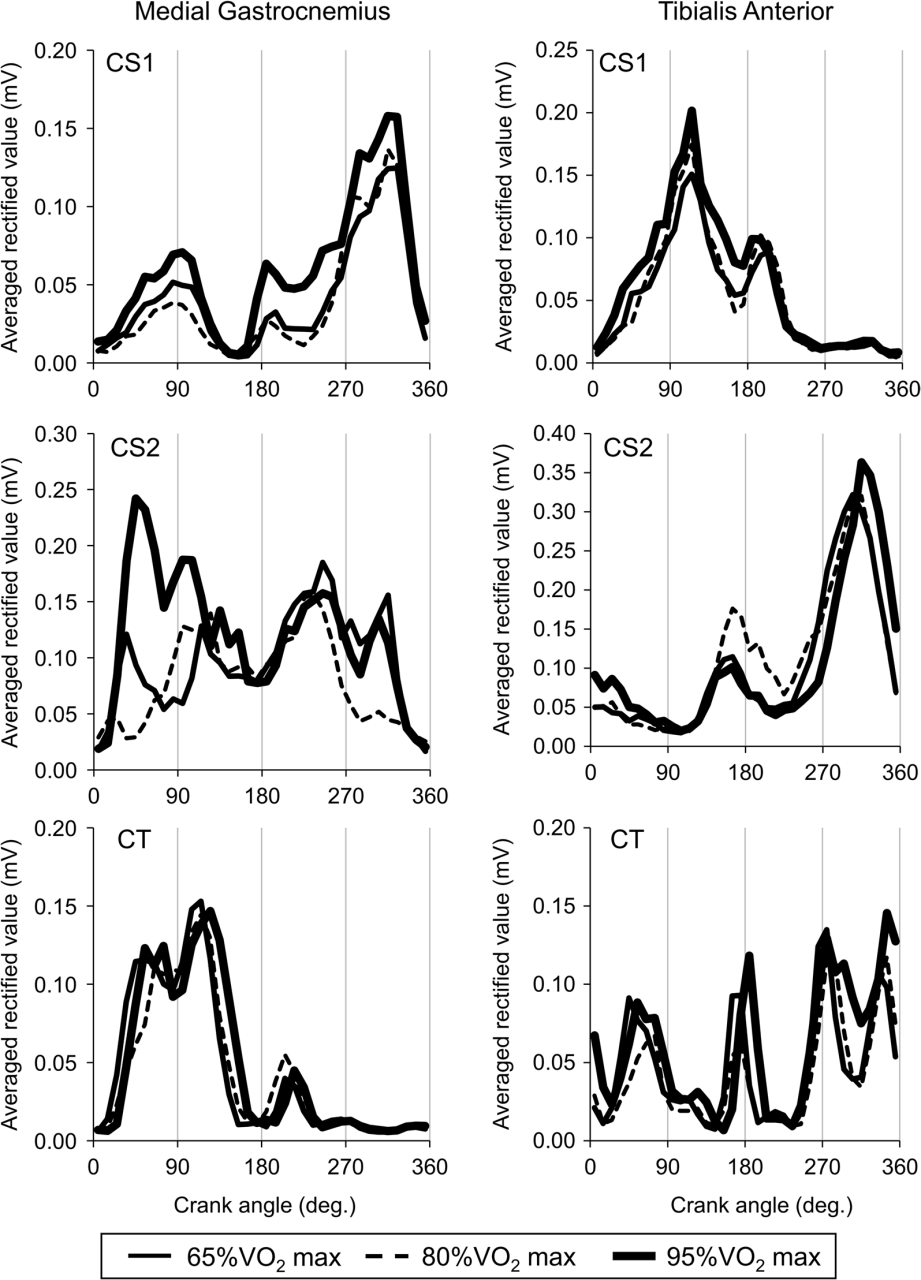
Averaged rectified values of surface electromyography during a crank cycle for the medial gastrocnemius muscle (Left panels) and the tibialis anterior muscle (Right panels) at various workloads. CS1 and CS2, cyclists with single amputation of leg; CT, cyclist with two legs

CS1 showed increases in ARV from 0° to 90° and 180° to 360° of crank angle and both activations were increased with an increase in workload (CS1 in left panels of Fig. 3). Increase in ARV for CS2 were found during a crank cycle (CS2 in left panels of Fig. 3).

#### Tibialis anterior (TA)

For CS1, increases in ARV were found from 0° to 180° of crank angle (CS1 in right panels of Fig. 3). CS2 showed increases in ARV around 180° of crank angle and from 270° to 360° of crank angle (CS1 in right panels of Fig. 3).

### Discussion

This regional neuromuscular activations along RF muscle were also shown in the present study, but CS1 and CS2 showed marked differences in neuromuscular activation patterns between RFp and RFd (Fig. 1). However, this difference were not found in CT (Fig. 1) and in non-cyclists in our previous study [11]. ARV of RFp increased during whole pulling phase in CS1 and CS2, while ARV of RFp increased during latter half of pulling phase in CT. It can be assumed that RFp for all participants contribute to hip flexion joint moment, but this action or joint torque would be emphasized in CS1 and CS2. This characteristic neuromuscular regulation in cyclists with single amputation of leg for hip flexion may be compensation to lack of hip and/or knee extension torque from contralateral leg. However, it should be noted that ARV of RFp during pulling phase were not increased with an increase in workload (Fig. 1). For example, ARV of VL (Left panels of Fig. 2) consistently increased with an increase in workload. This would reflect contribution to generate crank force for increasing workload. We thus estimated that neuromuscular activation in RFp could not contribute to generate crank force to increase workload and its act as pulling up their leg.

Activations of hamstrings muscles such as BF during pedaling movements is one of characteristic strategies in cyclists [10] and could contribute to pull up actions to rotate crank along the pedal trajectory. In the present study, CS1 and CS2 showed increases in ARV of BF during first half of pulling phase that was not found in CT (Right panels of Fig. 2). The previous study showed increase in surface EMG amplitude during pushing phase in cyclists [2, 9, 13]. This characteristic activation of BF in cyclists with single amputation of leg would contribute to knee flexion joint moment, since knee flexion joint torque is observed during latter half of pushing phase and first half of pulling phase [4]. [Hasson et al. [5]] also showed a small increase in sEMG for hamstrings muscles and knee flexion joint moment following one-leg pedaling practice [5]. However, similarly to ARV of RFp during pulling phase (Left panels of Fig. 1), ARV of BF during first half of pulling phase was also not increased with an increase in workload (Right panels of Fig. 2). This means BF also could not act as generator of crank force and its activation contributes to pulling up their leg during first half of pulling phase.

Gastrocnemius muscles act as planter flexor and knee flexor because of their anatomical properties. From planter flexion joint torque is observed during pushing phase and knee flexion joint torque is seen mainly during pulling phase [4], it can be estimated that increases in ARV during pushing phase and pulling phase are associated with planter flexion and knee flexion joint torque, respectively. In this study, increase in ARV of MG were found during pushing phase for all participant and during pulling phase for CS1 and CS2 (Left panels of Fig. 3). As similar with CT in this study (Left panels of Fig. 3), neuromuscular activation of MG were mainly found during pushing phase, but not during pulling phase [2, 9]. Therefore, we assumed that MG commonly acted as planter flexor for all participants in this study and this muscle also contributed to knee flexion joint moment in CS1 and CS2.

In conclusion, as we hypothesized, characteristic neuromuscular activation patterns were observed in cyclists with single amputation of leg for RFp, BF, and MG, which associate with hip and knee flexion moment, during pulling phase.

## Limitations

Number of the participants were limited. Further studies are needed to clarify the characteristic neuromuscular strategy in in cyclists with single amputation of leg. Also, we measured sEMG only from six muscles out of large number of lower extremity muscles. Therefore, we should note that other muscles also should contribute to generate crank rotation force in characteristic strategy during pedaling in cyclists with single amputation of leg.

## Data Availability

Data will not be shared and available for authors.

## Abbreviations

ARV: Averaged rectified values
BF: Biceps femoris
CS1 and CS2: Cyclists with single amputation of leg
CT: Cyclist with two legs
EMG: Electromyography
MG: Medial gastrocnemius
RF: Rectus femoris
RFd: Distal regions of rectus femoris
RFp: Proximal region of rectus femoris
TA: Tibialis anterior
VL: Vastus lateralis
VO_2_ max: Maximum oxygen consumption

## References

[1] Cannon DT, Kolkhorst FW, Cipriani DJ (2007). Effect of pedaling technique on muscle activity and cycling efficiency. Eur J Appl Physiol.

[2] Dorel S, Drouet JM, Couturier A, Champoux Y, Hug F (2009). Changes of pedaling technique and muscle coordination during an exhaustive exercise. Med Sci Sports Exerc.

[3] Gregor RJ, Broker JP, Ryan MM (1991). The biomechanics of cycling. Exerc Sport Sci Rev.

[4] Gregor RJ, Cavanagh PR, LaFortune M (1985). Knee flexor moments during propulsion in cycling-a creative solution to Lombard’s Paradox. J Biomech.

[5] Hasson CJ, Caldwell GE, van Emmerik RE (2008). Changes in muscle and joint coordination in learning to direct forces. Hum Movement Sci.

[6] Hermens H, Freriks B, Merletti R, Stegeman D, Blok J, Rau G, Disselhorst-Klug C, Hägg G (1999). European recommendations for surface electromyography.

[7] Hug F, Dorel S (2009). Electromyographic analysis of pedaling: a review. J Electromyogr Kinesiol.

[8] Hug F, Marqueste T, Le Fur Y, Cozzone PJ, Grelot L, Bendahan D (2006). Selective training-induced thigh muscles hypertrophy in professional road cyclists. Eur J Appl Physiol.

[9] Li L, Caldwell GE (1998). Muscle coordination in cycling: effect of surface incline and posture. J Appl Physiol.

[10] Takaishi T, Yamamoto T, Ono T, Ito T, Moritani T (1998). Neuromuscular, metabolic, and kinetic adaptations for skilled pedaling performance in cyclists. Med Sci Sports Exerc.

[11] Watanabe K, Kouzaki M, Moritani T (2015). Heterogeneous neuromuscular activation within human rectus femoris muscle during pedaling. Muscle Nerve.

[12] Watanabe K, Kouzaki M, Moritani T (2012). Task-dependent spatial distribution of neural activation pattern in human rectus femoris muscle. J Electromyogr Kinesiol.

[13] Watanabe K, Sato T, Mukaimoto T, Takashima W, Yamagishi M, Nishiyama T (2016). Electromyographic analysis of thigh muscles during track cycling on a velodrome. J Sports Sci.

